# Alcohol, Hormones, and Health in Postmenopausal Women

**Published:** 1994

**Authors:** Laura J. Tivis, Judith S. Gavaler

**Affiliations:** Laura J. Tivis, Ph.D., is a research associate at the Oklahoma Transplantation Institute, Baptist Medical Center of Oklahoma, Oklahoma City, Oklahoma. Judith S. Gavaler, Ph.D., is a member of the Oklahoma Medical Research Foundation; chief of the Women’s Health Research Program, Oklahoma Medical Research Foundation; and chief of women’s research, Oklahoma Transplantation Institute, Baptist Medical Center of Oklahoma, Oklahoma City, Oklahoma

## Abstract

Menopause is associated with increased risk for certain diseases. By affecting hormone levels, alcohol consumption might influence the occurrence or progress of these diseases.

Women today can expect to live one-third of their lives after menopause (Gavaler 1991). Moreover, a large proportion of postmenopausal women are at least moderate consumers of alcohol^1^ (Gavaler 1993*a*; Gavaler and Rosenblum 1994). The effects of alcohol on hormone levels in this large population are of interest for several reasons. First, the normal hormonal changes of menopause are associated with increased risk for certain diseases of the bones and heart. By affecting hormone levels, alcohol might influence the occurrence or progress of these diseases.

Second, alcohol-hormone interactions may influence the development of alcohol-induced liver disease in those postmenopausal women who are heavier drinkers.

Finally, postmenopausal women are the ideal female population in which to study the effect of alcohol on hormones in women. Before menopause, hormone levels fluctuate daily, depending on the stage of the menstrual cycle. After menopause, hormone levels remain constant (at their new postmenopausal levels). It is therefore easier to measure alcohol-induced changes in hormone levels in post- than in pre-menopausal women.

## Hormonal Regulation Before and After Menopause

Before examining the effects of alcohol, it will be helpful to review the hormonal regulation of the reproductive cycle and the changes that occur during a natural menopause. The female reproductive cycle is governed primarily by two groups of hormones: the gonadotropins–luteinizing hormone (LH) and follicle stimulating hormone (FSH)–and the estrogens. Every 28 days or so, the gonadotropic hormones cause follicles to develop in the ovaries. On the 14th day of the cycle, one of these follicles ovulates (i.e., produces an ova). Estrogen is secreted during the growth of the follicles; after ovulation, large amounts of estrogen are secreted from the secretory gland (corpus luteum) that replaces the follicle. As long as estrogen concentrations remain high, LH and FSH are inhibited and will not initiate development of additional follicles. Two weeks after ovulation, if pregnancy does not occur, the corpus luteum degenerates and the ovarian hormones (e.g., estrogen) decrease dramatically, LH and FSH no longer are inhibited, and menstration begins.

When a woman reaches approximately 40 to 50 years of age, her reproductive cycle usually becomes irregular, and ovulation does not take place during many of these cycles. During this time, few follicles still remain that can be stimulated by FSH and LH; and as the number of follicles decrease, the production of estrogens also decreases. When estrogen production falls below a certain threshold, the estrogens can no longer inhibit the production of the gonad-otropins and the oscillatory cycle stops, resulting in menopause.

The loss of signficant amounts of estrogen from the woman’s system often results in substantial physiological adjustments (e.g., “hot” flashes, fatigue, anxiety). Estrogens are commonly prescribed to alleviate these unpleasant symptoms or other adverse health consequences of menopause. This practice is known as estrogen replacement therapy.

However, the body’s production of estrogen does not cease entirely after menopause. Estrogens also can be produced from another class of hormones called androgens. Androgens are produced in the ovaries and in the adrenal glands located atop the kidneys. In men, androgens are the primary sex hormones; for example, the androgen testosterone is responsible for the development of the male characteristics, such as increased body hair and bone growth, and the development of spermatozoa. In women, the androgens have only a slight masculinizing effect. Instead, they travel through the bloodstream to body fat, where they are converted to estrogens through a process called aromatization.

## Alcohol’s Effects on Hormones After Menopause

Estrogen levels after menopause are influenced by factors that affect androgen production or aromatization. Because the ovaries are a source of androgens, women who have undergone surgical removal of the ovaries (ovariectomy) have less estrogen than women who have had a natural menopause. Aromatization occurs in fat tissue, so underweight women have lower levels of estrogen than do women who are overweight. Studies in men have shown that alcohol can not only stimulate the adrenal glands to make androgens but also can increase the conversion of androgens to estrogens (Gordon et al. 1975, 1976; Longcope et al. 1984). This suggests that alcohol consumption may increase estrogen levels in postmenopausal women as well.

The effects of moderate alcohol consumption on hormonal function were first reported in a study of 244 normal post-menopausal women from four countries (Denmark, Portugal, Spain, and the United States) (Gavaler et al. 1991). The term “normal” in this context indicates freedom from serious medical problems, such as cancer, heart disease, or liver disease. The average age of the women was 59.3 years, and the average time since menopause was 8.0 years; 80.6 percent were classified as having had a natural menopause, whereas an average of 19.4 percent had had ovariectomies. None of the women were taking estrogen replacement therapy. The characteristics of the women, grouped by country, are shown in table 1.

Moderate alcohol consumption for women has been defined as no more than one drink per day (U.S. Department of Agriculture, U.S. Department of Health and Human Services 1990). Of those women in table 1 who reported current use of alcohol, 68 percent consumed less than one drink per day. Levels of estradiol (the most potent natural estrogen) were measured in both the moderate alcohol drinkers and the abstainers. The moderate drinkers from all countries except Spain had significantly higher levels of estradiol compared with the levels in abstainers. These results suggest that alcohol, in moderate amounts, is associated with increased estrogen levels in postmenopausal women.

**Figure 1:**
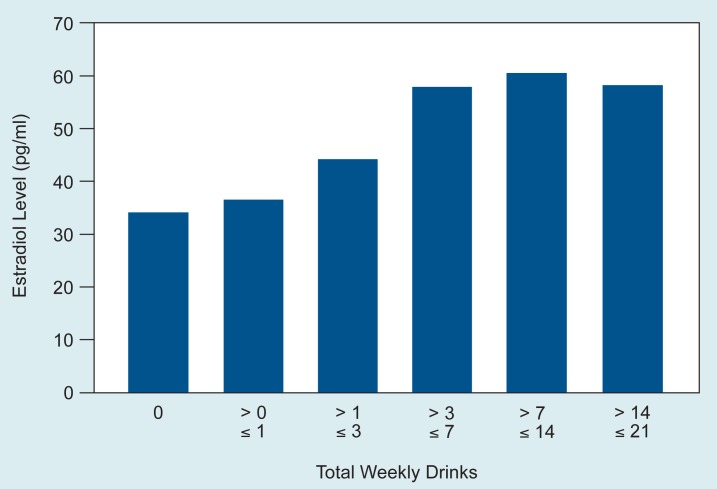
Estradiol levels of normal postmenopausal women from several countries. Estradiol levels remain stable when total alcohol consumption exceeds seven drinks per week. SOURCE: Gavaler et al. 1991.

Estrogen replacement therapy has been associated with decreased risk for coronary heart disease^2^ and osteoporosis,^3^ suggesting a protective role for estrogens in these diseases. Therefore, it is important to determine whether estrogen levels continue to rise with increasing alcohol consumption. Figure 1 shows that estradiol levels among the 244 study women remained stable when total alcohol consumption exceeded 7 drinks per week. These findings are consistent with data reporting a significantly reduced risk for coronary heart disease at a level of one drink per day (Klatsky 1990), suggesting that any benefit derived from increased estrogen is maximal when alcohol intake averages no more than one drink per day.

In the same study group, hormones other than estradiol also were increased by alcohol consumption, including the androgen testosterone and the pituitary hormone prolactin.^4^ The E_2_:T ratio (an estimate of the amount of testosterone converted to estradiol) also increased (Gavaler 1993*a*). FSH and LH decreased as a normal response to the increase in estrogen.

The variability in estrogen levels of women from different countries suggests that other unknown factors such as ethnic background or diet also might influence estradiol levels and that moderate alcohol consumption might have different effects on different populations. These results are of interest, given the current emphasis on special populations in alcohol research.

## Alcohol and Cirrhosis in Postmenopausal Women

Alcohol’s effects on hormones may affect the progress and treatment of alcohol-induced diseases. One of the most serious medical consequences of alcoholism is liver cirrhosis. Alcohol also has been found to increase estrogen levels in alcoholic postmenopausal women with cirrhosis (reviewed in Gavaler 1993*b*). In earlier studies, however, the number of women studied was small, and only limited conclusions could be drawn.

In a more recent study with more than 90 subjects, it was possible not only to compare hormone levels but also to evaluate hormone relationships and to determine whether hormone levels were related to the severity of alcohol-induced liver disease (Gavaler 1993*b*; in press). Table 2 compares a group of 66 alcoholic postmenopausal women with alcohol-induced cirrhosis with 27 normal alcohol-abstaining postmenopausal control subjects. The two groups of women did not differ statistically in terms of age, weight, body mass, or prevalence of ovariectomy. Liver disease severity was evaluated based on laboratory tests as well as clinical signs and symptoms. None of the women are taking estrogen replacement therapy. Compared with alcohol-abstaining controls, the alcoholic cirrhotic women had significantly elevated estradiol and prolactin levels and E_2_:T ratios as well as significantly lower levels of testosterone, LH, and FSH. These findings confirmed previous reports (see Gavaler 1993*b*).

In addition, the data from this study extended previous findings: relationships among hormones were substantially disrupted in the postmenopausal women with alcohol-induced cirrhosis. In the alcohol-abstaining subjects, the relationships were as expected: low levels of estradiol were related to high levels of LH and FSH, and increased body mass was related to increased levels of estradiol. In the alcoholic cirrhotic subjects, however, statistically significant correlations of estrogen levels with LH, FSH, and body mass were undetectable. The relationships between age and levels of LH and FSH also were examined. In the normal alcohol-abstaining control women, analysis revealed that as postmenopausal women became older, both LH and FSH levels decreased. Yet in the postmenopausal alcoholic cirrhotics, both LH and FSH increased with age (Gavaler in press).

Interestingly, statistical analyses suggested that LH, FSH, prolactin, estrogen, testosterone, and the E_2_:T ratio in the alcoholic cirrhotic women were disrupted in proportion to the severity of the cirrhosis. These results suggest that hormone relationships are affected by cirrhosis itself as well as by alcohol consumption. The significance of these results is unclear.

## Hormone Levels After Liver Transplantation

Liver transplantation is the only effective treatment for terminally ill patients with cirrhosis. Gavaler and coworkers (1993) studied the effect of liver transplantation on hormone levels in nine alcoholic postmenopausal women approximately 2 years after the surgery. Of these women, two reported having a drink one to two times a month and a third reported consuming two drinks per day, whereas the rest abstained. In the total sample of nine postmenopausal women, although levels of LH and FSH had returned to normal in the interval, levels of estradiol, testosterone, and prolactin, as well as the E_2_:T ratio, remained similar to levels in cirrhotic women before transplantation. Moreover, the normal levels of LH and FSH represent an abnormal hormonal relationship, given that estrogen levels remained elevated and unchanged following transplantation. The significance of these findings is unclear.

## Summary and Conclusions

Alcohol exerts notable effects on the hormone levels of postmenopausal women. Estradiol levels are significantly higher among postmenopausal women who drink moderately (one drink or less per day) compared with postmenopausal women who abstain. These findings are important, considering that only about 30 percent of postmenopausal women take estrogen replacement therapy (Harris et al. 1990) and that decreased estrogen levels have been associated with increased risk for certain diseases.

The effects of long-term heavy drinking on hormone levels are dramatic, including significant differences in the levels of estradiol, testosterone, prolactin, LH, and FSH, and the E_2_:T ratio. The normal pattern of relationships between estradiol and other factors was severely disrupted in the women with cirrhosis.

Unknown factors—such as race, ethnic group, or diet—may modulate alcohol’s effects in different groups of women. Furthermore, the degree to which cirrhosis itself affects hormonal relationships in alcoholic postmenopausal women is unclear; alcoholic postmenopausal women with and without alcohol-induced liver disease must be compared to address this issue. Much research remains to be done to understand alcohol’s effects on hormone levels and women’s health.

## Figures and Tables

**Table 1:** Characteristics of Normal Postmenopausal Women Grouped by Country of Origin

Characteristic	United States (Pittsburgh) *n* = 128	Denmark (Copenhagen) *n* = 62	Portugal (Lisbon) *n* = 34	Spain (Madrid) *n* = 20
Age (yr)	57.7 ± 0.4	64.0 ± 1.0	58.2 ± 1.3	57.1 ± 1.4
Alcohol Users (%)	79	95	31	75
Total Weekly Drinks	5.7 ± 0.6	6.9 ± 0.8	12.4 ± 2.6	5.4 ± 1.3
Among Alcohol Users				
Time Since Menopause (yr)	8.8 ± 0.5	14.8 ± 1.2	10.9 ± 1.6	8.2 ± 1.6
Prevalence of Ovariectomy (%)	27	5	26	20
Level of Estradiol^1^ (pg/ml)				
Abstainers	27.5 ± 3.6	35.1 ± 3.4	41.6 ± 2.3	46.7 ± 8.6
Alcohol users	44.3 ± 3.0	68.1 ± 2.5	89.0 ± 26.8	45.4 ± 5.8

1 The difference in estradiol levels between abstainers and alcohol users was significant for women in the United States, Denmark, and Portugal.

SOURCE: Gavaler et al. 1991.

**Table 2:** Characteristics and Hormone Levels of 66 Alcoholic Postmenopausal Women With Alcohol-Induced Cirrhosis and 27 Normal Alcohol-Abstaining Postmenopausal Control Women

Characteristic/ Hormone Level	Alcoholic Cirrhotic Women	Normal Control Women
Age (yr)	56.8 ± 1.2	57.7 ± 1.0
Weight (kg)	67.1 ± 1.8	66.4 ± 3.0
Body Mass Index^1^	24.8 ± 0.6	25.5 ± 5.7
Prevalence of Ovariectomy (%)	21.2	29.6
Estradiol (pg/ml)	62.7 ± 10.4	27.5 ± 3.3
Testosterone (ng/ml)	0.49 ± 0.05	0.74 ± 0.1
E_2_:T Ratio^2^	172 ± 27.0	44.5 ± 5.8
Luteinizing Hormone (miu/ml)	8.0 ± 1.4	24.0 ± 2.8
Follicle-Stimulating Hormone (miu/ml)	28.0 ± 4.4	63.3 ± 5.5
Prolactin (ng/ml)	16.4 ± 2.2	5.7 ± 0.4

1 A measure of height vs. weight.

2 The E_2_:T ratio is an estimate of the amount of testosterone converted to estradiol.

SOURCE: Gavaler 1993*b*.

## Footnotes

1 Throughout this article, terms such as “alcohol abuse,” “alcoholism,” “moderate drinking,” and “heavy drinking” are used. There is overlap among these terms in the alcoholism literature. Therefore, the wording in each case is based on the terms used in the reference cited unless otherwise specified.

2 Coronary heart disease involves decreased blood flow to the heart muscle; it may result in a heart attack, the most frequent cause of death in developed countries (Klatsky 1990).

3 Osteoporosis is a loss of bone tissue, leading to pain and increased risk of fracture.

4 The main function of prolactin is to stimulate milk secretion during pregnancy.
